# Supraphysiological Glucocorticoid Doses and Pitfalls of Annual Biomarker Monitoring in Adults With CAH

**DOI:** 10.1111/cen.70172

**Published:** 2026-06-18

**Authors:** Jakob Bolinder, Oskar Ragnarsson, Gudmundur Johansson

**Affiliations:** ^1^ Department of Endocrinology Sahlgrenska University Hospital Gothenburg Sweden; ^2^ Sahlgrenska Academy University of Gothenburg Gothenburg Sweden

1

Management of congenital adrenal hyperplasia (CAH) remains a balancing act between androgen excess and glucocorticoid overexposure. We performed a retrospective review of 58 adults with 21‐hydroxylase deficiency (21‐OHD) followed at the Department of Endocrinology at the Sahlgrenska University Hospital during the period 2020–2024. While the limited study size and retrospective design warrant cautious interpretation, the findings nonetheless point to important challenges in current practice.

The cohort included all forms of confirmed 21‐OHD (*n* = 58), predominantly classic CAH (salt‐wasting and simple virilizing; *n* = 50). Half of the patients (*n* = 29) used daily doses above 13 mg/m^2^/day, approximating the midpoint of the recommended dosing range in pediatric care (10–15 mg/m^2^/day). Furthermore, a total of 11 patients followed a reverse‐circadian regimen, most commonly with prednisolone at bedtime (*n* = 9) (Table 1). Treatment was individualized; the number of daily doses was influenced by glucocorticoid type, phenotype, and patient preferences, with long‐standing stable regimens sometimes maintained in the absence of clear adverse effects or unsatisfactory response to treatment changes. Although sick day rules and emergency care are addressed with patients, parenteral hydrocortisone rescue treatment was prescribed in only 27.6% overall and 36.4% of patients with salt‐wasting CAH, representing an important area for improvement.

**Table 1:** Glucocorticoid treatment regimens, adherence, and fludrocortisone treatment among adult patients with CAH, stratified by sex and genotype.

	Total	Female	Male	SW	SV	NC	Uncertain
	*n* = 58	*n* = 32	*n* = 26	*n* = 33	*n* = 17	*n* = 4	*n* = 4
**HCE dosage**, Mean (±SD)							
mg	22.47 (10.75)	**19.77 (10.23)**	**25.80 (10.63)**	**26.48 (8.84)**	**16.99 (10.67)**	10.63 (10.08)*	24.50 (10.68)
mg/m^2^ BSA	12.25 (4.93)	11.57 (6.09)	13.09 (2.85)	**13.99 (3.56)**	**9.24 (5.44)**	10.03 (5.10)	13.07 (7.70)
**Regimen**, *n* (% within group)							
No steroid treatment	3 (5.2)	2 (6.3)	1 (3.8)	0	2 (11.8)	1 (25.0)	0
Single dose	9 (15.5)	4 (12.5)	5 (19.2)	3 (9.1)	4 (23.5)	1 (25.0)	1 (25.0)
2‐dose	25 (43.1)	13 (40.6)	12 (46.2)	14 (42.4)	7 (41.2)	1 (25.0)	3 (75.0)
3‐dose	18 (31.0)	11 (34.4)	7 (26.9)	13 (39.4)	4 (23.5)	1 (25.0)	0
4‐dose	3 (5.2)	2 (6.3)	1 (3.8)	3 (9.1)	0	0	0
≥ 13 mg/m^2^	29 (50.0)	15 (46.9)	14 (53.8)	19 (57.5)	6 (35.3)	1 (25.0)	3 (75.0)
*Previously used*	17 (29.3)	7 (21.9)	10 (38.5)	10 (30.3)	6 (35.3)	0	1 (25.0)
*For different indications*	2 (3.4)	2 (6.3)	0	1 (3.0)	1 (5.9)	0	0
Reverse‐circadian dosing	11 (19.0)	5 (15.6)	6 (23.1)	7 (21.2)	4 (23.5)	0	0
*≥ ½ of dose afternoon*	4 (6.9)	2 (6.3)	2 (7.7)	3 (9.1)	1 (5.9)	0	0
*Bedtime prednisolone*	9 (15.5)	3 (9.4)	6 (23.1)	6 (18.2)	3 (17.6)	0	0
**Prescribed**, *n* (% within group)							
Hydrocortisone	49 (84.5)	28 (87.5)	21 (80.8)	29 (87.9)	13 (76.5)	3 (75.0)	4 (100)
*Dual/modified‐release*	3 (5.2)	0	3 (11.5)	2 (6.1)	1 (5.9)	0	0
Cortisone acetate	2 (3.4)	1 (3.1)	1 (3.8)	1 (3.0)	1 (5.9)	0	0
Prednisolone	10 (17.2)	4 (12.5)	6 (23.1)	8 (24.2)	2 (11.8)	0	0
*Previously used*	23 (39.7)	14 (43.8)	9 (34.6)	14 (42.4)	6 (35.3)	1 (25.0)	2 (50.0)
Combination HC/P	4 (6.9)	2 (6.3)	2 (7.7)	4 (12.1)	0	0	0
Fludrocortisone	37 (63.8)	18 (56.3)	19 (73.1)	**33 (100)**	**1 (5.9)**	0	3 (75.0)
*Previously used*	6 (10.3)	3 (9.4)	3 (11.5)	**—**	**6 (35.3)**	0	0
Solu‐Cortef	16 (27.6)	8 (25.0)	8 (30.8)	12 (36.4)	3 (17.6)	1 (25.0)	0

*Note:* Bold numbers denote statistical significance (*p* < 0.05) between male and female, or between genotypes. * = (*p* < 0.05) compared to SW genotype.

Abbreviations: BSA, calculated body surface area; HCE, hydrocortisone equivalent; HC/P, hydrocortisone/prednisolone.

In addition to controlling clinical hyperandrogenism in women and supporting fertility in both men and women, a central aim in the long‐term follow‐up of patients with CAH has been to reduce supraphysiological doses and to avoid non‐physiological regimens such as reverse‐circadian dosing. This is motivated by previous reports linking glucocorticoid overexposure in adults with CAH to obesity, hypertension, insulin resistance, and reduced bone mineral density [2], and by studies suggesting that evening cortisol elevations exert more detrimental metabolic effects than morning elevations [1].

Blood samples were mostly collected in the morning before each out‐patient visit without any instructions related to last glucocorticoid dose. Mean 17‐hydroxyprogesterone (17‐OHP) levels exceeded reference values in the majority of patients (*n* = 49). Testosterone in women, particularly in the presence of hirsutism or fertility concerns, and androstenedione in men may offer greater clinical utility [3]. These concentrations were frequently outside the reference intervals, as illustrated in Figure 1. Importantly, however, normalization of these biomarkers should not be the therapeutic goal in most patients, as they may instead lead to glucocorticoid excess and overtreatment [3]. Nevertheless, the same patient frequently presented with values both above and below the reference range at different time points, often in the context of dose adjustments and variations in sampling relative to dosing. This illustrates the limited reliability of these biomarkers in fine‐tuning dosing and in guiding long‐term disease control. Thus, annual “biochemical snapshots” provide only limited insight and should be interpreted with caution in clinical decision‐making.

**Figure 1:**
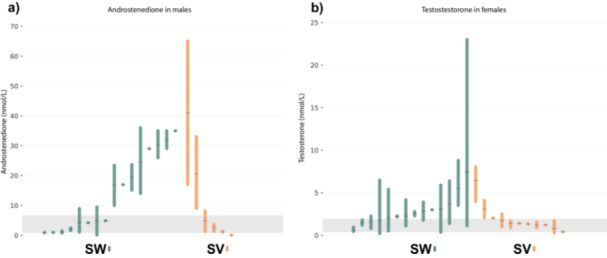
Measurements (conducted 2020–2024) of androstenedione in male (a) and testosterone in female (b) patients with 21‐hydroxylase deficiency. Each bar represents an individual patient with multiple measurements, where the lowest and highest values can be read. The lines within the bars indicate the patient's mean value. SW refers to the salt‐wasting subtype and SV refers to the simple virilizing subtype. The gray‐shaded area represents the reference range, simplified for all ages. The number of patients with missing data is 3.

Another observation was the frequent reevaluation of patients' presumed phenotype in adult care. Genotype–phenotype discrepancies have previously been reported [4], and similar findings were noted in our cohort. For example, six of 39 patients with a presumed salt‐wasting phenotype were reclassified during follow‐up as having a simple virilizing form, based on aldosterone and renin measurements. Following these reevaluations, all six patients discontinued fludrocortisone treatment. This may reflect the influence of modifier genes in CAH, and highlights the importance of viewing CAH phenotypes as a continuum rather than fixed categories, and of integrating genetic testing with measurements of renin and aldosterone to correctly assess mineralocorticoid requirements.

The challenges in current practice may improve with ongoing therapeutical development. Block‐and‐replace regimens, with drugs targeting the CRH, ACTH, or Melanocortin 2 receptors, are emerging as promising therapeutic strategies to suppress androgen excess that allow lowering glucocorticoid exposure. Yet, if androgen‐based markers lose relevance under these treatments, clinicians may still face the same fundamental difficulty as in other forms of adrenal insufficiency, that is, the absence of reliable biomarkers to guide and individualize cortisol replacement [5, 6]. Without such tools, the goal of physiological substitution will remain elusive.

In conclusion, our findings highlight two pressing needs in adult CAH care: refinement of therapeutic strategies to avoid chronic supraphysiological exposure, and the development of new, reliable biomarkers that can capture true cortisol requirements in daily life. Addressing both is essential if treatment is to move beyond current limitations and align more closely with physiological reality.

## Data Availability

The data that support the findings of this study are available from the corresponding author upon reasonable request.
